# *Notes from the Field:* Increase in Human and Animal Tularemia Cases — Minnesota, 2024

**DOI:** 10.15585/mmwr.mm7413a3

**Published:** 2025-04-17

**Authors:** Maria Bye, Jordan Mandli, Aaron Barnes, Elizabeth Schiffman, Kirk Smith, Stacy Holzbauer

**Affiliations:** ^1^Minnesota Department of Health Foodborne, Waterborne, Vectorborne, and Zoonotic Diseases Section; ^2^Minnesota Department of Health Public Health Laboratory; ^3^Career Epidemiology Field Office, CDC.

**Summary**

**What is already known about this topic?**

Tularemia is an uncommon but potentially serious bacterial zoonotic disease typically transmitted by an arthropod bite or exposure to infected animals. Although cases are reported from almost every state, tularemia is most frequently reported in the central United States. During 2000–2023, a median of one human and two animal tularemia cases were reported annually in Minnesota.

**What is added by this report?**

During 2024, five human and 27 animal tularemia cases were reported in Minnesota. All human patients were hospitalized; none died. Investigation of all human and animal cases did not definitively identify a source.

**What are the implications for public health practice?**

Tularemia should be considered in febrile patients with potential tularemia exposure and in animals with compatible signs or symptoms. Animal cases can be used to direct public health messaging to pet owners and others at higher risk for exposure.

Tularemia is an uncommon but potentially serious bacterial zoonotic disease caused by the U.S. Department of Health and Human Services Tier 1 select agent *Francisella tularensis* that affects 200–300 persons annually in the United States (*1*). Although cases are reported from almost every state, tularemia is most frequently reported from the central United States. Symptoms vary based on the exposure route and commonly include fever and localized symptoms (e.g., lymphadenopathy and skin ulcers). Humans and animals are usually infected through the bites of arthropods (typically ticks and deer flies), contact with infected animals, or inhalation of contaminated material. No Food and Drug Administration–approved vaccine for prevention of tularemia exists. During 2000–2023, a median of one human and two animal cases were reported annually to the Minnesota Department of Health (MDH) and Board of Animal Health, respectively. Animal tularemia cases increased significantly in 2023, when 20 animal cases, but no human cases, were reported. In 2024, five human cases and 27 animal cases were reported in Minnesota, all in the seven-county Minneapolis-St. Paul metropolitan area. MDH staff members investigated each reported human and animal case to identify exposures and provide public health recommendations. This activity was reviewed by CDC, deemed not research, and was conducted consistent with applicable federal law and CDC policy.*

## Investigation and Outcomes

MDH obtained medical records and interviewed tularemia patients to collect information on their illness and exposures and obtained veterinary records and interviewed animal owners to learn about tularemia-positive animal illnesses, identify exposures, and assess zoonotic exposures in the household. Exposures of clinical laboratory staff members were also assessed. Veterinary personnel were assessed for occupational exposures to tularemia-positive animals. A high-risk exposure to an infected animal was defined as a bite, scratch, needlestick injury, or exposure to aerosolized infectious material from an infected animal. Any person with a high-risk household, veterinary, or clinical laboratory exposure was recommended antibiotic postexposure prophylaxis (PEP), generally with doxycycline or ciprofloxacin (*2*). Site investigations (i.e., tick drag sampling and site characterization using a standardized data collection form) were conducted for animal cases with illness onset in the past month or any human case in the Minneapolis-St. Paul metropolitan area (after obtaining consent) (*3*).

### Human Cases and Human Exposures

In 2024, the MDH Public Health Laboratory identified five laboratory-confirmed human tularemia cases (all *F. tularensis* subsp. *tularensis*) (Table), among whom four patients had the pneumonic form of tularemia. During 2000–2023, among 32 tularemia cases identified in Minnesota, two (6.3%) were pneumonic tularemia. All five patients were hospitalized for a median 6 (IQR = 3) days. In three cases, tularemia was not identified as the cause of infection until after hospital discharge. Once tularemia was identified, patients were treated with oral ciprofloxacin or doxycycline, and all recovered without complication. Two clinical laboratorians manipulated cultures outside of a biosafety cabinet and were advised to self-monitor for tularemia signs or symptoms for 14 days; neither experienced illness.

**TABLE:** Characteristics of human and animal tularemia cases — Minnesota, 2024

Characteristic	No. (%)*
**Human patients (n = 5)**	
Female	2 (40)
White, non-Hispanic	5 (100)
Age, yrs, median (range)	64 (45–72)
**Disease attribute**	
Pneumonic	4 (80)
Ulceroglandular	1 (20)
Hospitalized	5 (100)
Hospitalization length, days (range)	6 (5–9)
**Signs and symptoms**	
Fever	5 (100)
Weakness and lethargy	5 (100)
Anorexia	4 (80)
Headache	3 (60)
Muscle aches	3 (60)
Cough	2 (40)
Altered mental status	2 (40)
Vomiting	1 (20)
Lymphadenopathy	1 (20)
Skin ulcer	1 (20)
Pericarditis	1 (20)
Encephalopathy	1 (20)
**Imaging results**	
Abnormal chest x-ray (i.e., pneumonia or pleural effusion)	4 (80)
**Underlying condition**	
Diabetes	2 (40)
**Exposure**	
Lawn mowing	4 (80)
Animal bite	1 (20)
**Outcome**	
Hospitalized	5 (100)
Length of hospitalization, days, median (range)	6 (5‒9)
Survived	5 (100)
**Animal cases (n = 27)**	
**Animal species**	
Feline	21 (78)
Canine	5 (19)
Lagomorph	1 (4)
**Disease attribute**	
Glandular	3 (11)
Oropharyngeal	10 (37)
Pneumonic	1 (4)
Typhoidal	11 (41)
Undetermined	2 (8)
**Likely transmission route**	
Animal contact	12 (44)
Animal ingestion	4 (15)
Vectorborne	1 (4)
Unknown	10 (38)
**Outcome**	
Survived	21 (78)
Died from tularemia infection	4 (15)
Euthanized	2 (7)
**Human exposures, no.**	
Pet owners	3
Veterinary staff members	1

* Percentage of total human or animal cases.

### Animal Cases

Twenty-seven tularemia-positive animals were reported by direct laboratory report or veterinarians via telephone or email in 2024. Twenty-one (78%) animal cases were in domestic cats, five (19%) in domestic dogs, and one (4%) in a wild rabbit. Typhoidal tularemia (nonlocalized infection) and oropharyngeal tularemia (fever, oral ulcers, and lymphadenopathy) were the most common clinical forms identified. Most pets (78%) survived, four (15%) died from their infection, and two (7%) were euthanized because of cost or prognosis. Three pet owners and one veterinary staff member were determined to have been exposed, and one pet owner was advised to receive PEP after a scratch from an infected cat. The pet owner took the course of PEP as advised. No cases of tularemia occurred among exposed pet owners.

Site investigations for three human and two animal cases were conducted at the likely exposure location and nearby public spaces. No ticks were found by drag sampling. Two patients with pneumonic disease who consented to a site investigation reported mowing over animal carcasses during their exposure period; a rabbit and mouse carcass were found at the likely exposure sites but were too decomposed for testing.

## Preliminary Conclusions and Actions

Compared with the median number of tularemia cases reported in Minnesota during 2000–2023, human tularemia cases increased from one to five and animal cases increased from two to 27 in 2024. Increased veterinary awareness after tularemia-related communications in 2023 likely contributed to the increase in animal tularemia case reporting, in addition to a true increase in cases. On June 20, MDH issued a press release to notify the public of the increased risk for tularemia in Minnesota (*4*). Veterinarian alert emails sent through the Minnesota Board of Animal Health veterinarian email distribution list and physician alerts provided on regularly scheduled infectious disease clinician calls raised awareness of the increase in cases.

Health care providers should consider tularemia in febrile patients with a history of tick or deer fly bites, contact with ill animals, or mowing over a rabbit or rodent, even in areas of the United States, such as Minnesota, where tularemia is less commonly reported. When ordering testing for a patient in whom tularemia is suspected, providers should alert the laboratory to ensure that laboratorians take appropriate precautions such as working in a biosafety cabinet and wearing gloves, gowns, and eye protection. Veterinarians should consider tularemia in cats and dogs with compatible symptoms, including high fever, oral ulcers, and lymphadenopathy. Although dogs are less likely to experience acute clinical illness than are cats, dogs might be at risk for severe infection (*5*). Persons who have higher-risk exposure to an animal with tularemia through bites, aerosolization, or direct contact with an animal’s body fluids, should promptly receive PEP (*2*). Animal cases can be used to direct protective public health messaging to pet owners and others at higher risk for exposure.

## Footnotes

* 45 C.F.R. part 46.102(l)(2), 21 C.F.R. part 56; 42 U.S.C. Sect. 241(d); 5 U.S.C. Sect. 552a; 44 U.S.C. Sect. 3501 et seq.
